# Differential Impact of Two Risk Communications on Antipsychotic Prescribing to People with Dementia in Scotland: Segmented Regression Time Series Analysis 2001–2011

**DOI:** 10.1371/journal.pone.0068976

**Published:** 2013-07-17

**Authors:** Bruce Guthrie, Stella A. Clark, Emma L. Reynish, Colin McCowan, Daniel R. Morales

**Affiliations:** 1 Primary Care Medicine, Population Health Sciences Division, Medical Research Institute, Dundee, Scotland, United Kingdom; 2 Primary Care, NHS Fife, Kirkcaldy, Fife, Scotland, United Kingdom; 3 Victoria Hospital, Kirkcaldy, Fife, Scotland, United Kingdom; 4 Robertson Centre for Biostatistics, Institute of Health and Wellbeing, University of Glasgow, Glasgow, Scotland, United Kingdom; 5 Population Health Sciences Division, Medical Research Institute, Dundee, Scotland, United Kingdom; Chiba University Center for Forensic Mental Health, Japan

## Abstract

**Background:**

Regulatory risk communications are an important method for disseminating drug safety information, but their impact varies. Two significant UK risk communications about antipsychotic use in older people with dementia were issued in 2004 and 2009. These varied considerably in their content and dissemination, allowing examination of their differential impact.

**Methods:**

Segmented regression time-series analysis 2001–2011 for people aged ≥65 years with dementia in 87 Scottish general practices, examining the impact of two pre-specified risk communications in 2004 and 2009 on antipsychotic and other psychotropic prescribing.

**Results:**

The percentage of people with dementia prescribed an antipsychotic was 15.9% in quarter 1 2001 and was rising by an estimated 0.6%/quarter before the 2004 risk communication. The 2004 risk communication was sent directly to all prescribers, and specifically recommended review of all patients prescribed relevant drugs. It was associated with an immediate absolute reduction in antipsychotic prescribing of 5.9% (95% CI −6.6 to −5.2) and a change to a stable level of prescribing subsequently. The 2009 risk communication was disseminated in a limited circulation bulletin, and only specifically recommended avoiding initiation if possible. There was no immediate associated impact, but it was associated with a significant decline in prescribing subsequently which appeared driven by a decline in initiation, with the percentage prescribed an antipsychotic falling from 18.4% in Q1 2009 to 13.5% in Q1 2011. There was no widespread substitution of antipsychotics with other psychotropic drugs.

**Conclusions:**

The two risk communications were associated with reductions in antipsychotic use, in ways which were compatible with marked differences in their content and dissemination. Further research is needed to ensure that the content and dissemination of regulatory risk communications is optimal, and to track their impact on intended and unintended outcomes. Although rates are falling, antipsychotic prescribing in dementia in Scotland remains unacceptably high.

## Introduction

Regulatory risk communications of various kinds are an important way of ensuring that prescribers are informed about new evidence of drug benefit and harm that emerges post-licencing. The impact and effectiveness of regulatory risk communications is highly variable though, with a systematic review of studies of the impact of US Food and Drugs Administration (FDA) risk communications finding that impact appeared to vary with the nature and specificity of the warning [1]. For example, recommendations to monitor treatment more closely had little impact whereas recommendations to avoid use in particular patient subgroups often did lead to reductions in use, especially if risk communications stated specific actions prescribers should take [1]. Although risk communications can therefore change prescribing, effects are variable and it is unclear how best to design or disseminate them [1], [2].

Antipsychotic drug use in older people with dementia has been the subject of several regulatory risk communications since 2002 [3]–[6]. Antipsychotic drugs are frequently prescribed with the aim of reducing behavioural and psychological symptoms of dementia (BPSD) in older people. In Scotland in 2007, 17.7% of people with a diagnosis of dementia were prescribed an antipsychotic [7], compared to approximately 12% in 2005–2007 in one US study [8]. Despite this high rate of use, antipsychotics have only limited benefit in treating BPSD in older people with dementia and carry significant risk of harm [9]–[12]. In 2009, antipsychotics were estimated to cause approximately 1800 deaths and 1620 cerebrovascular events in people with dementia in the UK annually [13]. However, clinical trial evidence in nursing home patients with dementia indicates that chronically prescribed antipsychotic drugs can be safely discontinued in most patients, with longer term follow-up suggesting a significant reduction in mortality [14] [15].

In the UK two main risk communications have been disseminated by the Medicines and Healthcare products Regulatory Agency (MHRA). The first was issued in March 2004, and highlighted newly discovered risks of stroke and death due to risperidone and olanzapine. It was directly and urgently disseminated to all prescribers, and contained explicit and clear guidance on how prescribers should respond (table 1) [3]. The second was issued in March 2009 and emphasised that these risks were associated with all antipsychotics. It was primarily disseminated in a limited circulation bulletin, with no explicit guidance on how prescribers should respond beyond being cautious in initiation (table 1), [16] although there were a number of other related guidance issued at around the same time. [5], [13], [17], [18] The aim of this study was to assess the impact of the 2004 and 2009 risk communications on antipsychotic and other psychotropic drug prescribing to older people with dementia in Scotland.

**Table 1:** 2004 and 2009 risk communications concerning antipsychotic use in older people with dementia.

Riskcommunication	Statement of risk(bold as in original text)	Advice on action (bold as in original text)
March 2004 riskcommunication(sent in a letter toall healthcareprofessionalsmarked “Urgentmessage”) [3]	“The CSM* has advised that there isclear evidence of an increased risk ofstroke in elderly patients withdementia who are treated withrisperidone or olanzapine. Themagnitude of this risk is sufficient tooutweigh likely benefits in thetreatment of behavioural disturbancesassociated with dementia and is acause for concern in any patient with ahigh baseline risk of stroke.”	**“Prescribing advice: CSM has advised that risperidone or olanzapine should not be used for the treatment of behavioural symptoms of dementia. Use of risperidone for the management of acute psychotic conditions in elderly patients who also have dementia should be limited to short-term and should be under specialist advice (olanzapine is not licensed for management of acute psychoses). Prescribers should consider carefully the risk of cerebrovascular events before treating any patient with a previous history of stroke or transient ischaemic attack. Consideration should also be given to other risk factors for cerebrovascular disease including hypertension, diabetes, current smoking and atrial fibrillation.** Although there is presently insufficient evidence to include other antipsychotics in these recommendations, prescribers should bear in mind that a risk of stroke cannot be excluded, pending the availability of further evidence. Studies to investigate this are being initiated. **Patients with dementia who are currently treated with an atypical antipsychotic drug should have their treatment reviewed.** Many patients with dementia who are disturbed may be managed without medicines. Treatment guidelines are available at websites listed below.”
March 2009 riskcommunication inDrug SafetyUpdate (limitedcirculationbulletin) [16]	**“Advice for healthcare professionals:**There is a clear increased risk ofstroke and a small increased risk ofdeath when antipsychotics (typical oratypical) are used in elderly peoplewith dementia.”	“The balance of risks and benefits associated with risperidone treatment should be carefully assessed for every patient, taking into consideration the known increased mortality rate associated with antipsychotic treatment in the elderly. Prescribers should carefully consider the risk of cerebrovascular events before treating with risperidone any patient who has a previous history of stroke or transient ischaemic attack. Consideration should also be given to other risk factors for cerebrovascular disease including hypertension, diabetes, smoking, and atrial fibrillation.”

* CSM = Committee for Safety of Medicines.

## Materials and Methods

### Ethics Statement

National Health Service Research Ethics Committee (NHS REC) review was not required because all data management and analysis only used anonymised data and was carried out consistent with the PCCIU standard operating procedures which have themselves been approved by the NHS Grampian Research Ethics Committee. All analysis was carried out using PASW Statistics v18 (IBM Software 2009).

### Population Studied

The population studied was patients aged 65 and over permanently registered with 87 Scottish general practices which contributed data for the entire period to a dataset held by the Primary Care Clinical Information Unit (PCCIU), University of Aberdeen. All participating practices consented to research use of anonymised data at the time of data extraction in Spring 2011. Data were extracted for all patients with a diagnosis of dementia at any point between 1^st^ January 2001 and 31^st^ March 2011. Dementia was defined either as the presence of a Quality and Outcomes Framework (QOF) defined dementia Read Code (used to define disease registers under the UK National Health Service contract for GPs) [19], an NHS Scotland Information Services Division defined dementia Read Code [20], or if the patient had ever having been prescribed an anticholinesterase inhibitor drug (defined as drugs listed in British National Formulary [BNF] section 4.11). Since antipsychotics are indicated in some older people with dementia and psychosis, people with QOF-defined ‘severe and enduring mental illness’ (predominately schizophrenia and related psychoses or severe bipolar disorder) were excluded from analysis. A quarterly time-series analysis was created, where individuals were included in analysis for each quarter if they were aged 65 years and over *and* had a dementia diagnosis at the beginning of the quarter.

### Outcomes

In each quarter, eligible patients were defined as being prescribed a particular drug class if they received one or more relevant prescriptions in that quarter. The drug classes studied were oral antipsychotics (drugs in BNF chapter 4.2.1), hypnotics (BNF 4.1.1), anxiolytics (BNF 4.1.2) and antidepressants (BNF 4.1.3), and the outcomes measured were the receipt of one or more relevant prescriptions for each drug class in any particular quarter. Two additional outcomes were defined. Antipsychotic initiation was defined as a patient receiving an antipsychotic in a particular quarter when there had been no antipsychotic prescription in the 6 months before the date of issue. Antipsychotic discontinuation was defined as a patient who had received an antipsychotic in the previous quarter but not in the current quarter.

### Statistical Methods

Time series for the specified outcomes were plotted and the impact of the two pre-specified regulatory risk communications examined in a single segmented regression analysis model, which is a form of interrupted time series analysis commonly used to evaluate policy interventions [21]. This method estimates three key parameters for each intervention: a) the slope or *trend* in prescribing before the intervention; b) the *change in the level* of prescribing immediately following the intervention; and c) the *change in trend* from the pre-intervention trend.

The dates chosen for the intervention were pre-specified as the date of dissemination of the two regulatory risk communications, which in both cases was at the end of the first quarter of the relevant year. Quarter 2 in 2004 and 2009 were therefore defined as the first post-risk communication time-point. The minimum number of people with dementia being measured in any time point was 1912, and the analysis was weighted for the number of patients with dementia included in each time point. The presence of serial autocorrelation was tested for in each model using the Durbin-Watson statistic and the Breusch-Godfrey test, but was not found to be significant in any model. Where appropriate, seasonal effects were accounted for by fitting fixed effects for ‘quarter’ as an independent variable using Aikake’s Information Criteria to select the best fitting model. Only main effects are presented in the paper.

## Results

Between 2001 and 2011, the total number of patients aged 65 years and over rose from 76,506 to 82,497 with the largest relative increases in the over-85s. The number of patients recorded as having dementia increased from 1912 (prevalence 2.5% of over 65 year olds, 95% CI 2.4–2.6) in quarter 1 2001 to 3478 (4.2%, 95% CI 4.1–4.4) in quarter 1 2011, which was only partially explained by the rise in the total number of people aged 65 and over, and the very elderly in particular (the dementia prevalence in quarter 1 2011 directly standardised to the quarter 1 2001 population structure was 3.8% (95% CI 3.7–3.9)). There were no changes in the rising trend in the prevalence of dementia around the times of the risk communications in 2004 and 2009. Across the entire period, approximately 1% of over-65s were excluded because they had a ‘severe and enduring mental illness’ diagnosis. The majority of people with dementia were women across the whole time period (75.8% in quarter 1 2001 and 68.9% in quarter 1 2011).

**Figure 1:**
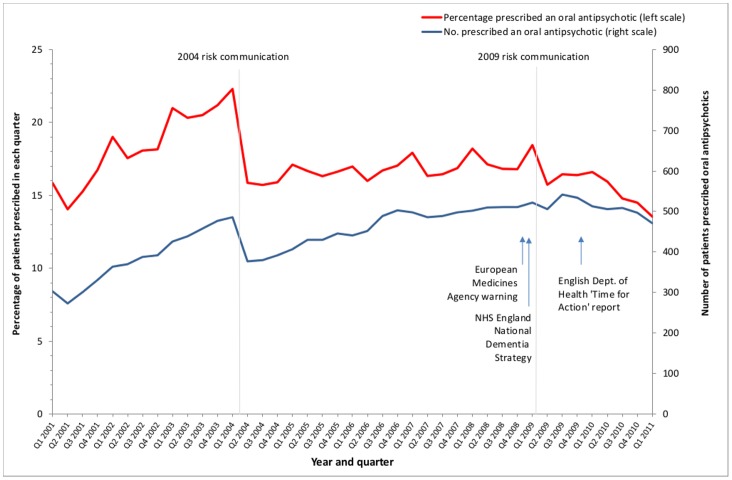
Prescribing of all oral antipsychotics in people aged ≥65 years with dementia.

Figure 1 shows time trends in the percentage of patients with recorded dementia prescribed any antipsychotic, with segmented regression analysis results for any antipsychotic prescription in table 2. In the segmented regression model, for all antipsychotics, there was a significantly rising trend in antipsychotic prescribing before the 2004 risk communication of 0.61% (95% CI 0.53 to 0.68) absolute increase per quarter from a model estimated baseline of 13.9% (table 2). The 2004 risk communication was associated with a large immediate absolute fall in antipsychotic prescribing of −5.94% (95% CI −6.64 to −5.23), with a downward change in trend of −0.54% per quarter (95% CI −0.63 to −0.45) afterwards. The overall effect was therefore of a large immediate drop in prescribing, with a change from a steadily rising trend (an additional 0.61% of people with dementia are prescribed an antipsychotic every quarter) to a flat one (0.61% minus 0.54% = 0.07% increase per quarter). In contrast, the 2009 risk communication was not associated with any immediate reduction in total antipsychotic prescribing, but there was a statistically significant change in trend of −0.51% (95% CI −0.64 to −0.37) per quarter in absolute rates of prescribing, equating to a shift from a flat to a falling trend.

**Table 2:** Segmented regression analysis of changes in antipsychotic and other psychotropic prescription in relation to the 2004 and 2009 risk communications.

	Baseline quarter1 2001 (intercept)% (95% CI)	Trend before2004 riskcommunication% (95% CI)	Change in levelafter 2004 riskcommunication% (95% CI)	Change in trendafter 2004 riskcommunication*% (95% CI)	Change in levelafter 2009 riskcommunication% (95% CI)	Change in trendafter 2009 riskcommunication*% (95% CI)
Oral antipsychoticprescribed	13.89 (13.24 to 14.53)	0.61 (0.53 to 0.68)^b^	−5.94 (−6.64 to −5.23)^b^	0.54 (−0.63 to −0.45)^b^	0.06 (−0.72 to 0.84)	−0.51 (−0.64 to −0.37)^b^
Oral antipsychoticinitiated	3.18 (2.47 to 3.89)	0.04 (−0.04 to 0.13)	−0.74 (−1.34 to −0.14)^a^	0.03 (−0.11 to 0.06)	−0.10 (−0.73 to 0.53)	−0.17 (−0.28 to −0.06)^a^
Oral antipsychoticdiscontinued	2.75 (1.92 to 3.58)	−0.06 (−0.16 to 0.03)	1.04 (0.24 to 1.84)^a^	0.01 (−0.12 to 0.10)	0.03 (−0.82 to 0.88)	0.08 (−0.06 to 0.23)
Hypnotic prescribed	8.63 (8.06 to 9.20)	0.02 (−0.05 to 0.09)	1.37 (0.75 to 2.00)^b^	0.08 (−0.15 to 0.002)	0.51 (−0.18 to 1.20)	−0.25 (−0.37 to −0.13)^b^
Anxiolytic prescribed	2.76 (2.24 to 3.27)	0.14 (0.08 to 0.21)^b^	1.32 (0.76 to 1.89)^b^	0.02 (0.09 to 0.05)	0.45 (−0.17 to 1.07)	−0.37 (−0.47 to −0.26)^b^
Antidepressant prescribed	17.19 (15.74 to 18.63)	0.71 (0.53 to 0.88)^b^	1.78 (0.20 to 3.36)^a^	0.18 (−0.37 to 0.02)	0.47 (−1.28 to 2.21)	−0.69 (−0.99 to −0.38)^b^

a p<0.05;

b p<0.001.

* Value is the *change* in trend not the subsequent trend, and interpretation of the model should be in conjunction with examining the time trend graphs. For example, for oral antipsychotics the trend before the 2004 intervention is a rising one, with an increase of 0.61% per quarter. There is a statistically significant downward change in trend of 0.54% per quarter, so the post-2004 risk communication estimated trend is an increase of 0.07% per quarter. There is a further statistically significant downward change in trend of 0.51% per quarter after the 2009 risk communication, so the post-2009 risk communication estimated trend is a decrease of 0.44% per quarter.

**Figure 2:**
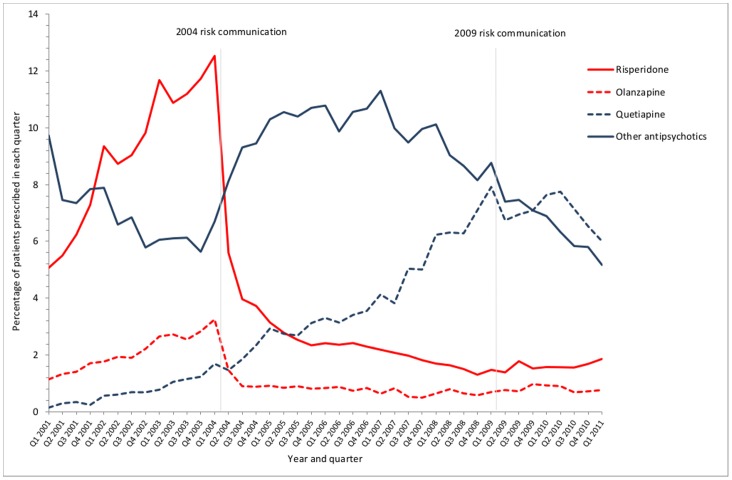
Prescribing of selected oral antipsychotics in people aged ≥65 years with dementia.

Figure 1 additionally shows the absolute number of people with recorded dementia prescribed an antipsychotic. Although the immediate changes and changes in trend are broadly mirrored in the absolute numbers prescribed, there were more people with recorded dementia prescribed an antipsychotic in 2011 than in 2001, reflecting that recorded prevalence had increased. Time trends for individual drugs show that prescribing of the two drugs specifically warned against (risperidone and olanzapine) fell rapidly in the quarter immediately after the 2004 risk communication, with partial replacement with other antipsychotics, predominately haloperidol initially (figure 2).

**Figure 3:**
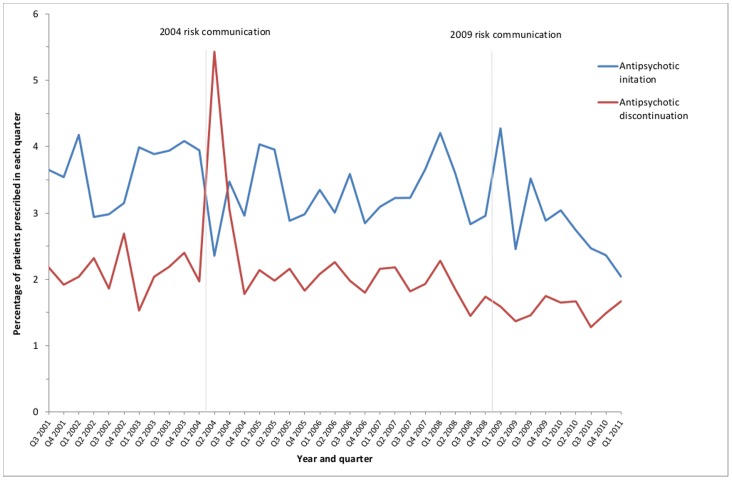
New antipsychotic prescribing and antipsychotic stopping in people aged ≥65 years with dementia.

**Figure 4:**
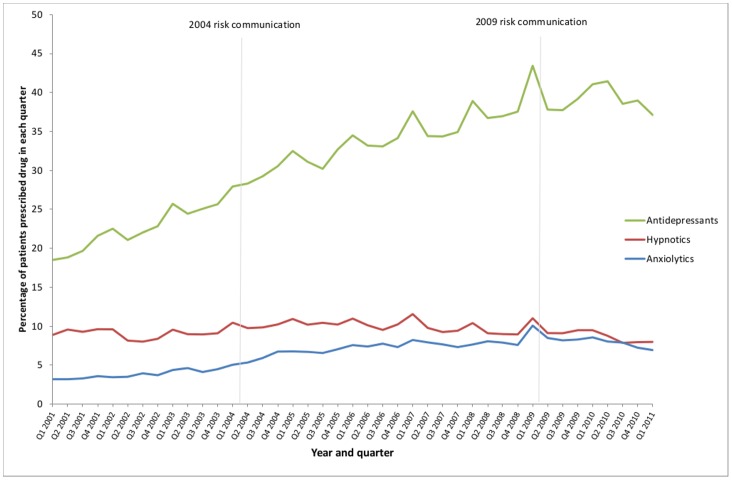
Hypnotic, anxiolytic and antidepressant prescribing in people aged ≥65 years with dementia.

The 2004 risk communication was associated with a transient decrease of −0.74% (−1.34 to −0.14) in antipsychotic initiation, without any statistically significant change in trend. In contrast, the 2009 risk communication was not associated with any immediate change, but there was a downward change in trend of −0.17% (95% CI −0.28 to −0.06) (table 2, figure 3). For antipsychotic discontinuation, there was a statistically significant transient increase immediately after the 2004 risk communication, but no subsequent change in trend, and no significant change of any kind following the 2009 risk communication (table 2, figure 4).

Time trends in prescribing of other psychotropic drugs are shown in figure 4, with segmented regression results in table 2. The 2004 risk communication was associated with transient absolute increases in hypnotic, anxiolytic, and antidepressant prescribing of 1.37% (95% CI 0.75 to 2.00), 1.32% (0.76 to 1.89) and 1.78% (0.20 to 3.36) among patients age 65 and over with dementia respectively. Hypnotic prescribing was static before 2004, with anxiolytic and antidepressant prescribing both significantly increasing, but there was no significant change in trends in any of the three drug classes in association with the 2004 risk communication. The 2009 risk communication was not significantly associated with any immediate change in prescribing of any of the three drug classes, but was associated with significant decreases in trend of −0.25% per quarter for hypnotics, −0.37% per quarter for anxiolytics and −0.69% per quarter for antidepressants.

## Discussion

### Summary of Findings

Although causality cannot be definitively ascribed, both the 2004 and 2009 MHRA risk communications were associated with statistically significant changes in antipsychotic prescribing. However the magnitude and patterns of change associated with each risk communication differed significantly. The 2004 risk communication was associated with an immediate large fall in the level of antipsychotic prescribing and a moderate change in the trend which was rising before it and flat after it. There was an associated decrease in both antipsychotic initiation and increase in antipsychotic discontinuation. In contrast, the 2009 risk communication was not associated with any immediate change in antipsychotic prescribing, but was associated with a change in trend from flat to falling of a similar magnitude to 2004. This was associated with a decline in antipsychotic initiation, with no evidence of any change in antipsychotic discontinuation. There was no evidence of associated significant substitution with other psychotropic drugs after either risk communication, and the 2009 risk communication was associated with significant downward changes in the trend for all three drug classes. While there did not appear to be immediate substitution, it is notable that antidepressant prescribing doubled over the 10 years examined (a greater increase than in general population antidepressant use over the period 1997–2010 [22]), although this trend flattened after 2009.

### Strengths and Limitations of the Analysis

The study used routine healthcare data which allowed the analysis of a long time series in a large dataset, but suffers the limitations that all such studies do in terms of the data potentially being incomplete because it was collected for another purpose. A particular issue is that dementia is known to be under-recorded historically (although Scottish recording is reasonably close to epidemiological predictions) [23]. The quarter 1 2011 dementia prevalence in this study was 4.2% in people aged 65 and over, compared to estimates of 6.6% and 6.4% from the largest UK study and an Europe-wide meta-analysis respectively [24]. However, the age-standardised prevalence of dementia in people aged 65 years and over increased from 2.5% in quarter 1 2001 to 3.8% in quarter 1 2011, and as figure 1 shows there were more people with a recorded diagnosis of dementia being prescribed an oral antipsychotic in 2011 than in 2001. Similar changes in recorded prevalence of dementia were seen in the Veteran’s Administration study by Kales et al [8], and there were no step changes in prevalence around the time of the risk communications that could explain the findings, particularly with regards the immediate impact of the 2004 risk communication. A second issue is that the study does not have data on reasons for antipsychotic prescribing, and so cannot examine the perceived indication for antipsychotic initiation, continuation or stopping. Although the data is consistent with the risk communications leading to a change in prescribing practice and the study design is as rigorous a method as can be used in the absence of randomisation [21], it is not possible to definitely ascribe causation to the observed association.

### Comparison with Other Studies

Three North American studies have examined the impact of regulatory risk communications on antipsychotic prescribing [8], [25], [26]. In Canada, three regulatory risk communications in the period 2002–2005 reduced the rate of growth of antipsychotic prescribing in people with dementia and caused some shift from risperidone and olanzapine to quetiapine [25], but total antipsychotic prescribing in older people continued to increase [27]. Two US studies of the impact of the 2005 FDA risk communications showed falls in antipsychotic use in older people with dementia [8], [26], but there was little immediate impact on the scale observed in the study reported here in association with the 2004 risk communication. To our knowledge, there are no published studies of subsequent regulatory risk communications in this field. Kales et al’s study in the Veterans’ Administration population also examined the use of other psychotropics, finding no change in hypnotic, anxiolytic or antidepressant use [8]. In contrast, our study shows that antidepressant prescribing rose considerably over the whole period. Although we found some evidence of transient substitution of other psychotropics for antipsychotics in 2004, the more striking finding was that prescribing of hypnotics, anxiolytics and antidepressants either flattened off or declined after the 2009 risk communication. This highlights that regulatory risk communications may have unexpected effects beyond the prescribing targeted, and evaluation should ideally seek to examine unintended as well as intended consequences [28], [29].

The NHS England national prescribing audit published in July 2012 showed a reduction in the proportion of older people with recorded dementia prescribed an antipsychotic from 17.0% in 2006 to 6.8% in 2011, [30] compared with the observed reduction in this study from 16.9% in quarter 1 2006 to 13.5% in quarter 1 2011. In England, the 2009 risk communication was reinforced by a Department of Health commitment to reduce antipsychotic prescribing in older people with dementia by two-thirds over two years [13] [18]. In contrast, there was no such clear policy response in NHS Scotland. The greater observed fall in antipsychotic prescribing in England is consistent with there being an additional impact of the policy response over and above the risk communication directed at the whole UK. However, it is important to note that the number of people with recorded dementia in the English audit more than doubled since 2006, compared with an ∼33% increase in the Scottish data examined in this analysis over the same period (reflecting Scotland’s better historical recording of dementia in GP records [23]). Changes in rates of antipsychotic use over time have to be treated with caution because of the shifting denominator of ‘recorded dementia’.

### Interpretation of the Findings

In an observational design of this nature, it is not possible to definitively ascribe causality to the statistical associations seen in segmented regression models of the kind used here. However, the 2004 risk communication was associated with a large change in prescribing consistent with the nature of the warning disseminated urgently to all prescribers (table 1). On the background of previously rising trends in the use of both, risperidone and olanzapine prescribing more than halved in the quarter following the risk communication (from 12.5% of older people with dementia to 5.6% for risperidone, and from 3.3% to 1.5% for olanzapine), with only partial immediate replacement by other antipsychotics. Our interpretation is that the 2004 risk communication prompted widescale review of people with dementia prescribed antipsychotics, with large changes in prescribing.

Interpretation of the impact of the 2009 risk communication is more ambiguous. There was no immediate change in antipsychotic prescribing, although we observed a statistically significant decline in antipsychotic use subsequently. This reduction in antipsychotic use was associated with a decline in initiation, was consistent with the 2009 risk communication which only highlighted caution in initiation as a specific action for prescribers (table 1). However, it is important to note that other publications at around the same time also highlighted concern about antipsychotic use in older people with dementia, including the European Medicines Agency report in December 2008 that prompted the 2009 risk communication, [5] the English National Dementia Strategy in February 2009, [17] and the English Department of Health ‘Time for Action’ report about antipsychotic use in older people with dementia published in November 2009 [13] (although the latter two did not strictly speaking apply in Scotland, they may still have affected practice). It is therefore possible that the observed statistically significant association between the 2009 risk communication and changes in antipsychotic prescribing is spurious. Our interpretation is that the impact of the 2009 risk communication was small at best, in contrast with the changes associated with the 2004 risk communication.

Although causality cannot be proven, our interpretation is that the data is consistent with the two risk communications having an impact which reflected differences in the nature and dissemination of the two risk communications. The 2004 risk communication made very explicit statements of the magnitude of risk, had specific recommendations to avoid, review and stop named drugs, and was urgently disseminated directly to all prescribers. In contrast, the 2009 risk communication made a less clear recommendation to be cautious in initiation, did not explicitly recommend review or stopping, and was disseminated via a limited circulation routine bulletin (table 1).

While it is impossible to know what the ‘right’ level of antipsychotic prescribing in older people with dementia is, it is notable that large numbers of older people with dementia continue to be treated with antipsychotics. Such prescribing is often in response to the need to manage distressing behavioural and psychological disturbance. Given the lack of highly effective alternative treatments, the correct level of antipsychotic use in this population is unlikely to be zero, although it is almost certainly less than current levels in Scotland [13], [31].

### Implications of the Findings

This study provides further evidence that risk communications from regulators do change clinical practice, although it raises important questions about how such risk communications should best be designed and disseminated [1]. Although an observational study cannot definitively ascribe causality, we believe that the 2004 risk communication was associated with a large change in prescribing, including a large initial impact most likely because it prompted widescale review of patients already being prescribed antipsychotics. While the 2009 risk communication was not associated with any immediate change in prescribing, the rates of prescribing subsequently fell (although whether this is due to the risk communication or other publications and policy activity around the same time cannot be determined in an analysis with pre-specified interventions to examine). The limited dissemination of the 2009 risk communication is of particular note. There were seven other risk communications sent directly to healthcare professionals in the UK in the first quarter of 2009, relating to efavirenz, temsirolimus, toremifine, bevacizumab, efalizumab, recombinant coagulation factor VIII, and fondaparinux [32]. Additionally, there was a large campaign to publicise new advice about the risks of over the counter preparations for colds and coughs in children [33]. Despite being more urgently and more widely disseminated, none of these were associated with the scale of harm of antipsychotics in dementia, which in 2009 were estimated to kill approximately 1800 people in the UK, and to additionally cause a further 1620 cerebrovascular events [13]. We therefore believe that the effectiveness of regulatory risk communications could be improved by better attention to the content and method of dissemination of risk communications, tailored to the level of risk and harm involved. Based on the larger observed changes in prescribing associated with the 2004 risk communication, and the smaller observed changes in 2009 despite multiple policy publications as well as the risk communication, it is also plausible that a clear and authoritative recommendation to review patients with dementia prescribed antipsychotics disseminated directly to prescribers would lead to wide-scale, targeted review and significant implementation of the guidance to stop antipsychotics wherever possible.

### Conclusions

This analysis provides evidence that risk communications from regulators did reduce antipsychotic prescribing in older people with dementia, but the observational design means that it is not possible to definitively ascribe any changes in prescribing with the warning. However, the evidence for impact is much stronger for the 2004 than the 2009 risk communication, consistent with the marked differences in their design and method of dissemination. Although impact in this kind of real-world intervention is likely to vary with context, the findings are consistent with previous research examining the impact of FDA risk communications in terms of impact varying with the design of the warning, and in particular of warnings clearly specifying the actions expected of prescribers having greater impact [1]. There is a need for applied research to systematically examine why impact varies in order to understand how better to design and disseminate regulatory risk communications to maximise effectiveness, and to routinely monitor the impact of risk communications on both intended and unintended consequences.
